# Efficacy of *Liriope platyphylla* extract on improving respiratory function: A CONSORT-randomized, double-blind, placebo-controlled pilot trial

**DOI:** 10.1097/MD.0000000000030073

**Published:** 2022-09-02

**Authors:** Eun Sol Won, Yong Ho Ku, Eun Yi Lee, Il-Woung Kim, Hyun Lee, Jae Hui Kang

**Affiliations:** a Department of Acupuncture & Moxibustion Medicine, College of Korean Medicine Daejeon University, Daejeon, Republic of Korea; b Department of Acupuncture & Moxibustion Medicine, Cheonan Korean Medicine Hospital of Daejeon University, Cheonan-si, Republic of Korea; c Department of New Business Team/Bio-research Center, D&L Biochem, Company-Affiliated Research Institute, Chungju-si, Republic of Korea; d Department of Future Convergence Industry, Bio Industry Team, Sejong Technopark, Sejong-si, Republic of Korea.

**Keywords:** breathlessness, cough, and sputum scale, Liriope platyphylla, pulmonary function, respiratory function

## Abstract

The respiratory system is the first line of defense against outside pollutants. Recently, respiratory health has been receiving increasing attention due to the increase in fine dust, which reduces respiratory function and increases incidence of chronic obstructive pulmonary disease, and in coronavirus pandemic, which can cause severe acute respiratory syndrome.

**Methods:**

This clinical pilot trial was designed to secure evidence for a main clinical trial and to confirm the efficacy and safety of *Liriope platyphylla* (LP) extract for improving respiratory function. We conducted a double-blind randomized placebo-controlled trial with 22 participants from June 30, 2021, to August 25, 2021. The primary outcome was Breathlessness, Cough, and Sputum Scale score. Secondary outcomes included forced vital capacity, forced expiratory volume at 1 second (FEV1), forced expiratory volume at 1 s/forced vital capacity ratio, cough assessment test score, chronic obstructive pulmonary disease assessment test score, peripheral blood mononuclear cell counts (white blood cells, eosinophils, T cells, and B cells), high-sensitivity C-reactive protein level, erythrocyte sedimentation rate, cytokine (interleukin-1β, interleukin-4, tumor necrosis factor-α, interleukin-6, interleukin-8, interferon-γ, and immunoglobulin E) levels, antioxidant (glutathione peroxidase and superoxide dismutase) levels, and nitric oxide level.

**Results:**

A total of 22 participants were randomly assigned to 2 groups: the LP group (n = 11), who took 1000 mg of LP extract per day, and the placebo group, who took 1000 mg of dextrin per day. Participants took 1 capsule twice a day for 4 weeks. For the Breathlessness, Cough, and Sputum Scale, the interaction between group and visit was statistically significant in a blend of analyses of variance. interleukin-8, tumor necrosis factor-α, and interferon-γ levels decreased more in the LP group than in the placebo group. The sample size required for large-scale clinical trials in the future was 50. There were no side effects.

**Conclusion:**

LP extract can enhance respiratory function. The detailed data we obtained support conducting the future main large-scale clinical trial.

## 1. Introduction

Respiratory diseases have emerged as a global issue due to coronavirus disease-19, which began to spread at the end of 2019. Accordingly, there is an emergent need for health supplement products for respiratory diseases^[1]^: as interest in respiratory function increases and interest in health supplements also increases. *Liriope platyphylla* (LP) is a food traditionally used to improve respiratory function and has been shown to be effective at improving lung capacity in dyspnea patients.^[2]^ LP increases mucin 5AC production and the levels of mucin 5AC gene expression in NCI-H292 cells. Mucin is an important component of mucus that contributes to its viscoelasticity. It also works with the cilia to remove harmful elements in the airway.^[3]^ Published in 2020, the Republic of Korea’s Guidance on New Functional Evaluation of Health Functional Foods states that studies concerning respiratory function enhancement should include biomarkers such as pulmonary function, inflammatory cells, cytokines, and intrabronchial antioxidants. There have been studies on the effect of LP on antioxidants and immune function, but there has not yet been a clinical trial with those biomarkers.^[4,5]^ We conducted a double-blind randomized controlled trial to test the efficacy and safety of LP for improving respiratory function as functional supplements. This pilot trial is the preparatory stage for a main clinical trial. The results of this trial will provide basic data for future large-scale clinical trials.

## 2. Materials and Methods

### 2.1. Study design

This investigator-initiated, single-center, double-blind, placebo-controlled study examined the efficacy and safety of LP extract for improving respiratory function over 4 weeks. This trial was registered with the Korean National Clinical Research Information Service (CRIS-KCT0006426) and was approved by the institutional review board (IRB) of Daejeon University Cheonan Korean Medicine Hospital (DJUMC-2021-BM-02-1). The study was performed according to the Declaration of Helsinki, the guidelines of good clinical practice, and standard operating procedures. A 4-week visit schedule was planned for the participants, and this trial was expected to have better compliance than other clinical trials. The clinical trial protocol ver1.0 and case report form ver1.0 were approved by the IRB, and the clinical trial protocol ver1.1, which was revised to correct typographical errors and to comply with a request from the IRB, was approved on April 19, 2021. All modifications were applied following IRB approval.

### 2.2. Protocol and sample size calculation

This pilot clinical trial was designed to confirm the efficacy of LP extract for improving respiratory function as a double-blinded randomized placebo-controlled trial. To perform a large-scale clinical trial, information regarding the basic safety evidence and sample count calculations must be obtained prior to the actual trials. This is a pilot study designed to find the basis of sample size calculation and feasibility for the main clinical trials. The sample size was determined empirically based on achieving 80% power and a moderate effect size. According to the suggestions of Whitehead et al,^[6]^ 20 candidates were planned for, with 10 in each group. Assuming a dropout rate of 5%, the final sample size required was 22. Twenty-five participants enrolled from May to June 2021 at DJUMC. All participants who met the inclusion criteria and not the exclusion criteria were enrolled and signed an informed consent form before initiation of the study.

### 2.3. Inclusion and exclusion criteria

The inclusion criteria were as follows: between 19 and 80 years old; existing persistent respiratory symptoms such as coughing or phlegm (Breathlessness, Cough, and Sputum Scale [BCSS] score of at least 1); and a ratio of forced expiratory volume at 1 s (FEV1) to forced vital capacity (FVC) of more than 70%. The exclusion criteria were as follows: respiratory disease with underlying pulmonary parenchymal destruction; pneumonia or tuberculosis; bronchial asthma, chronic obstructive pulmonary disease, influenza, or lung cancer; acute or chronic bronchitis (BCSS score of 9 or more); uncontrolled cardiovascular disease; treatment with adrenocortical hormones, immunosuppressants, health supplements, or cough medication within four weeks; renal failure; liver dysfunction; uncontrolled psychopathy or alcoholism; participation in other clinical studies within one month; and pregnancy or lactation.

### 2.4. Randomization and blinding

The participants who met the criteria were assigned in a 1:1 ratio to the LP or placebo group using block randomization. An independent statistician generated the random allocation table and randomly set the size of the block. The total number of random allocations was generated at 120% of the target recruitment population. A 3-digit identification code was assigned to each participant according to the randomized allocation history. Randomization numbers were generated by statisticians using nQuery Advisor 7.0, Statistical Analysis System 9.0, or statistical package for the social sciences 21.0, and delivered to the manufacturer. The randomized number and group-assignment details are placed in a nonpermeable, sealed bag. This was given to the principle investigator for storage and management. The appearance and labeling of the LP extract and placebo capsules were identical. Both the participants and the investigators were blinded to the group allocation of the participants. Each participant was assigned their 3-digit identification code by the investigator according to the order of their enrollment, and this identification code was used to distribute the capsules to the participants. If a participant dropped out, their randomization number was not used again. The group allocation of the participants was sealed and managed by the principal investigator and was not disclosed until the end of the clinical trial. In the event of serious adverse effects, only the affected participant’s envelope would be opened to confirm the details of their group allocation. Both investigator and subjects were blinded through blinding procedure.

### 2.5. Intervention

Participants were randomly allocated to 1 of the 2 groups and received treatment accordingly for 4 weeks. Participants took 500 mg of either LP extract or dextrin twice per day at specific times. Clinic visits were scheduled at the end of the 4-week period to assess the efficacy and safety of the treatment. The LP capsules (LP extract, 500 mg) and placebo capsules (dextrin, 500 mg) were made by D&L Biochem (Chungju, Republic of Korea) and Hankookshinyak Pharmaceutical (Nonsan, Republic of Korea), respectively. The 2 capsules had the same size, appearance, color, and weight.

### 2.6. Objective and outcomes

This clinical trial was designed to confirm the efficacy and the safety of LP for improving respiratory function as functional supplements. The subjects were healthy individuals with respiratory problems without any underlying diseases. Hence, the questionnaires related to the subjective discomfort were mainly evaluated, and the immune indices, inflammatory cytokines, and antioxidant effect were additionally evaluated. The primary outcome was BCSS score. Secondary outcomes were FVC, FEV1, and FEV1/FVC, where FVC is the amount exhaled after breathing in as much as possible and then exhaling fully, and FEV1 is the amount exhaled within the first second. Additional secondary outcomes were cough assessment test (COAT) score; chronic obstructive pulmonary disease assessment test (CAT) score; pulmonary function test (PFT) score; peripheral blood mononuclear cell counts, including white blood cell, eosinophil, T cell, and B cell counts; high-sensitivity C-reactive protein (hsCRP) level; erythrocyte sedimentation rate (ESR); cytokine levels, including interleukin-1β (IL-1β), interleukin-4 (IL-4), tumor necrosis factor-α (TNF-α), interleukin-6 (IL-6), interleukin-8 (IL-8), interferon-γ (IFN-γ), and immunoglobulin E (IgE), which play an essential role in inflammatory reactions and infections; and antioxidant levels, including glutathione peroxidase (GPx), superoxide dismutase (SOD), and nitric oxide (NOX) levels. Outcomes were measured at baseline and 4 weeks.

### 2.7. Data analysis

Statistical analysis was performed for the full analysis set (FAS) population. The Mann-Whitney-Wilcoxon rank-sum test for continuous variables, Fisher exact test for categorical variables, and analyses of variance were conducted to compare demographic variables between the LP and placebo groups. The main analysis method was FAS, but the per-protocol group was also analyzed as a reference. The Mann-Whitney-Wilcoxon rank-sum test was used to analyze the average rank sum of BCSS score changes. Treatment differences between the LP and placebo groups were derived from the Hodges-Lehmann estimate and 95% nonparametric confidence interval. If the distribution of changes for each group satisfied normality, a linear mixed-effects model for repeated measures was used to confirm the trend in BCSS score changes. Continuous data such as laboratory tests and biological signs were analyzed using a paired or independent *t* test compared to the baseline. Categorical data were analyzed using a chi-squared test and Fisher exact test to confirm differences between the groups. Continuous data were also analyzed using a paired or independent *t* test. The analysis was evaluated using a 95% confidence interval, and the significance level of statistical analysis was α = 0.05 on both sides. When there were missing data, a last observation carried forward analysis was used.

### 2.8. Withdrawal and dropout

Research participants would have dropped out from the trial in the following cases: at their own request; occurrence of a serious adverse event; major violation of the protocol; use of prohibited drugs; treatment with medicines or health supplements that may have affected the clinical trial; and pregnancy. In each dropout case, the reasons had to be recorded in detail in the case report form. Participants who dropped out also underwent clinical laboratory tests to evaluate safety. If an adverse event occurred, follow-up observation would proceed until the cause had been identified. The results would be reported based on safety evaluation criteria and methods. In the case of a serious adverse event, the IRB would be notified immediately.

## 3. Results

**Figure 1.**
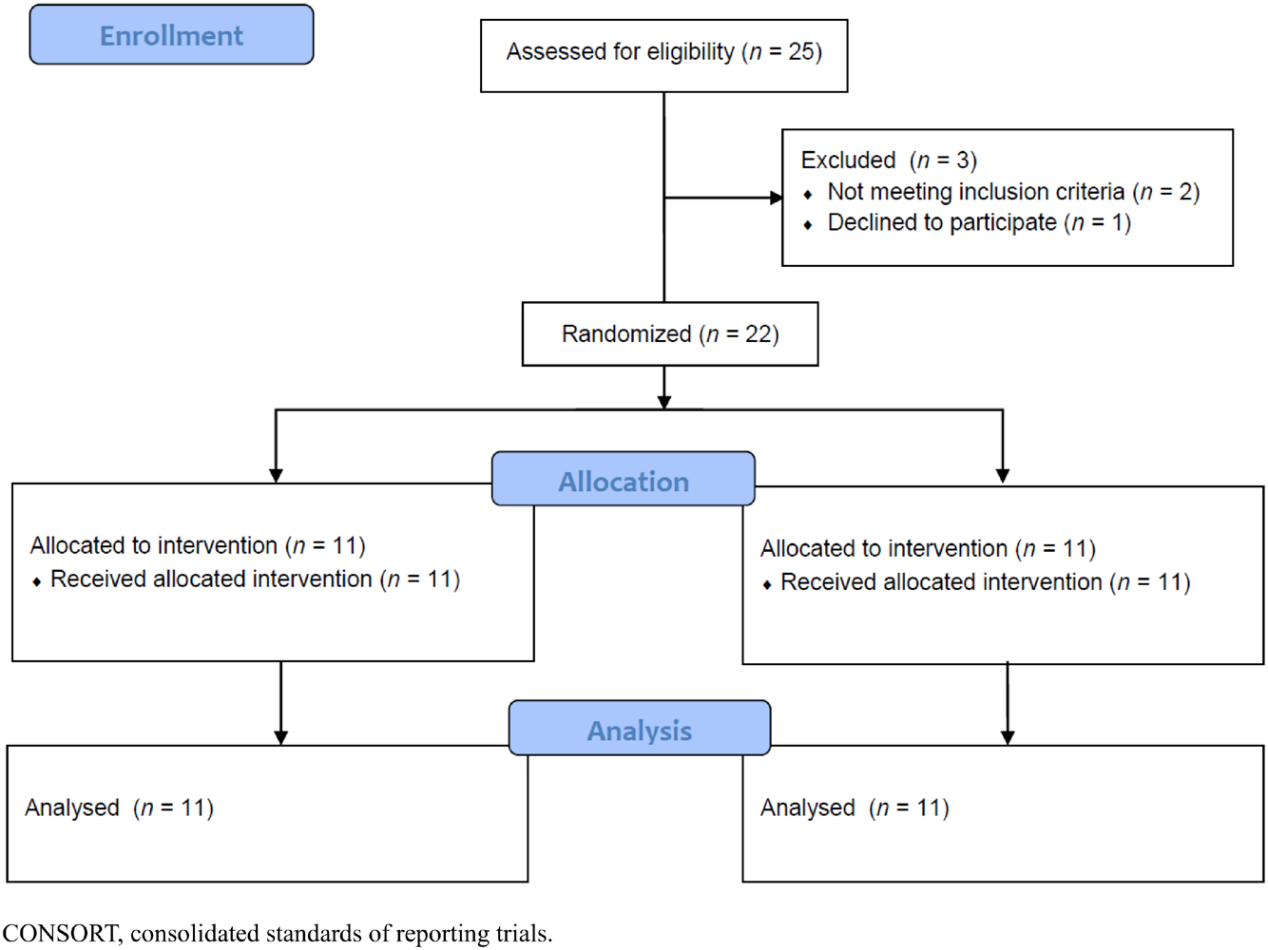
CONSORT 2010 flow diagram. CONSORT = consolidated standards of reporting trials.

From June 30 to August 25, 2021, 25 participants volunteered for this clinical trial, 22 of whom were randomly assigned 1:1 to either the LP (n = 11) or the placebo (n = 11) group. These 22 participants completed the trial, and there were no significant differences in demographic and clinical characteristics between the groups (Fig. 1 and Table 1). None of the participants dropped out. The FAS population consisted of a total of 22 participants.

**Table 1:** Participant characteristics at baseline.

Characteristic	Placebo, *n* = 11	LP, *n* = 11	*P* *
Sex: female, *n* (%)	9 (82%)	10 (91%)	>.999
Age (yr), mean (SD)	51 (10)	51 (12)	.531
Height (cm), mean (SD)	161.1 (5.2)	161.7 (7.4)	.793
Weight (kg), mean (SD)	57 (9)	58 (9)	.974
BMI (kg/m²), mean (SD)	21.70 (2.33)	21.95 (2.35)	.948
Systolic BP (mm Hg), mean (SD)	119 (11)	118 (12)	.974
Diastolic BP (mm Hg), mean (SD)	78 (9)	73 (10)	.236
Pulse (bpm), mean (SD)	76 (8)	77 (11)	.669
Body temperature (°C), mean (SD)	36.67 (0.29)	36.70 (0.36)	.505
Alcohol, *n* (%)			
None	7 (64%)	9 (82%)	.635
Moderate	4 (36%)	2 (18%)	
Heavy	0 (0%)	0 (0%)	
Smoking, *n* (%)			
None	9 (82%)	10 (91%)	>.999
Previous	1 (9.1%)	1 (9.1%)	
Current	1 (9.1%)	0 (0%)	
Day until EOF (visit 2) (d)			
Mean (SD)	28.18 (1.66)	29.55 (2.50)	.072
Median (IQR)	28.00 (27.50, 28.00)	28.00 (28.00, 31.00)	
Min–max	27.00–33.00	27.00–34.00	
Compliance (%)			
Mean (SD)	96.7 (3.3)	94.8 (6.9)	.972
Median (IQR)	96.4 (94.6, 100.0)	100.0 (90.0, 100.0)	
Min–max	90.9–100.0	82.1–100.0	
Hypertension, *n* (%)	2 (18%)	1 (9.1%)	>.999
Hyperlipidemia, *n* (%)	0 (0%)	1 (9.1%)	>.999
Diabetes, *n* (%)	0 (0%)	1 (9.1%)	>.999
Osteoporosis, *n* (%)	1 (9.1%)	1 (9.1%)	>.999
Misc., *n* (%)	2 (18%)	2 (18%)	>.999

BMI = body mass index, BP = blood pressure, EOF = end of file, IQR = inter quartile range, LP = Liriope platyphylla, SD = standard deviation.

* Categorical variable: Fisher exact test; Continuous variable: Wilcoxon rank-sum test.

**Figure 2.**
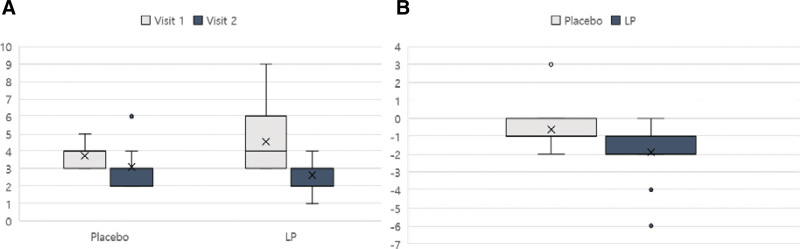
Breathlessness, Cough, and Sputum Scale (BCSS) score for both groups. (A) BCSS score at each visit in each group. (B) Change in BCSS score from the baseline. BCSS = Breathlessness, Cough, and Sputum Scale, LP = *Liriope platyphylla*.

**Figure 3.**
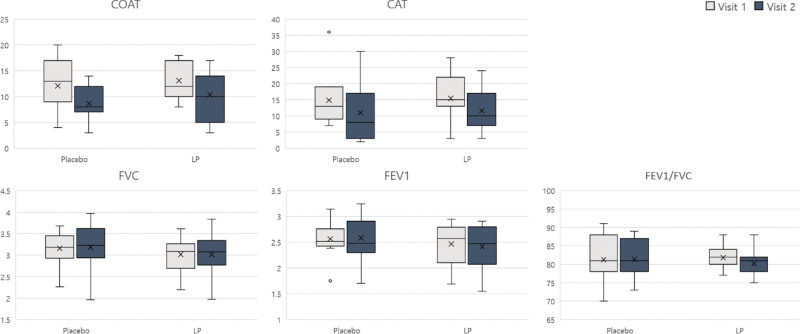
Secondary outcomes: questionnaires and pulmonary function test (PFT). CAT = chronic obstructive pulmonary disease assessment test, COAT = cough assessment test, FEV1 = forced expiratory volume at 1 s, FVC = forced vital capacity, LP = *Liriope platyphylla.*

**Figure 4.**
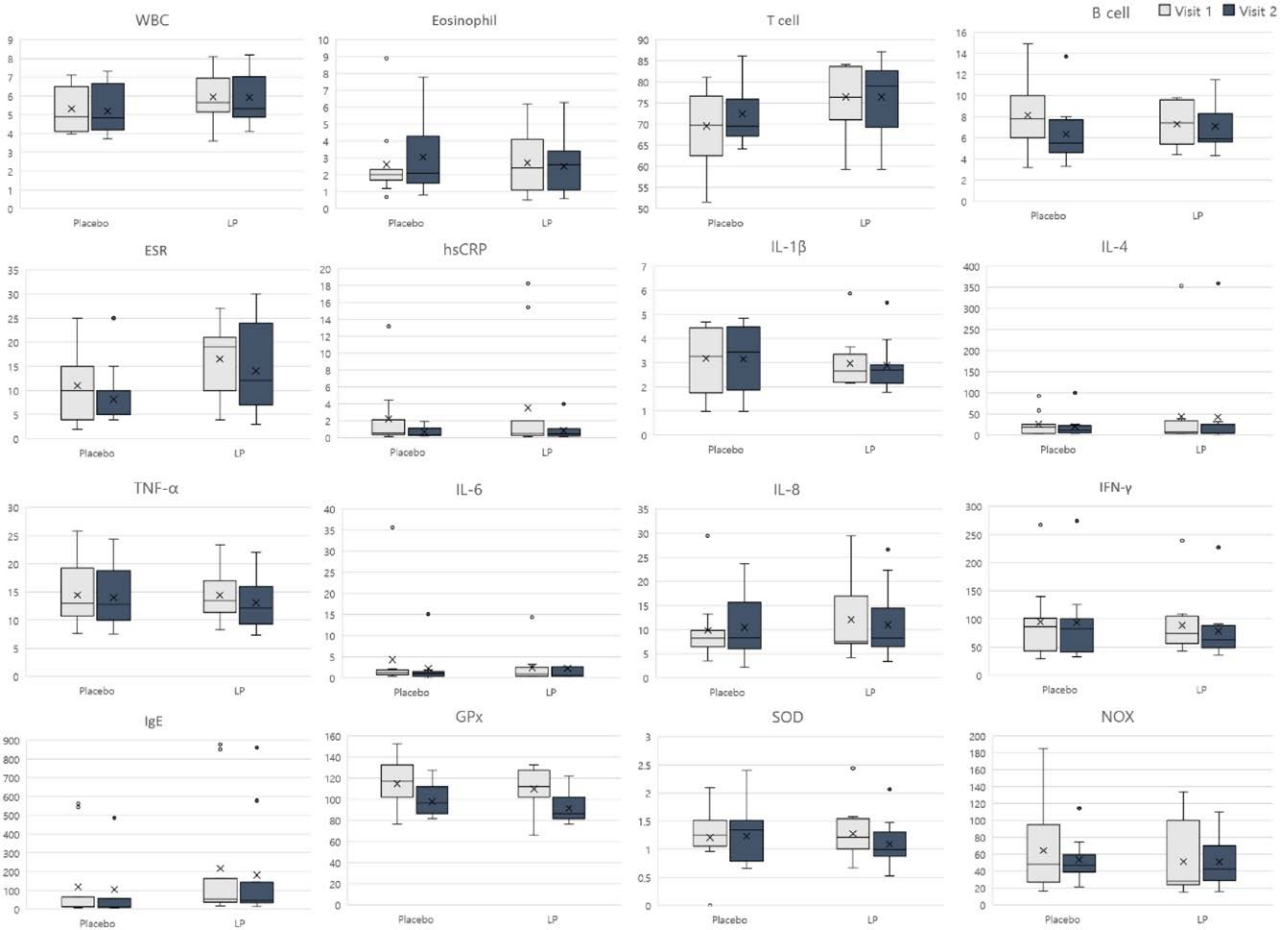
Secondary outcomes: laboratory test. ESR = erythrocyte sedimentation rate, GPx = glutathione peroxidise, hsCRP = high-sensitivity C-reactive protein, IFN-γ = interferon-γ, IgE = immunoglobulin E, IL-1β = interleukin-1β, IL-4 = interleukin-4, IL-6 = interleukin-6, IL-8 = interleukin-8, LP = Liriope platyphylla, NOX = nitric oxide, SOD = superoxide dismutase, TNF-α = tumor necrosis factor-α, WBC = white blood cells.

The primary outcome, represented with the BCSS score, was decreased in both placebo and LP groups (Fig. 2A). However, the change in BCSS score was greater in the LP group compared to the control group (Fig. 2B) In terms of the secondary outcomes, PFT, the COAT and CAT scores showed no significant differences (Fig. 3 and Table 2). The immune indices (white blood cells, eosinophils, T cells, B cells, hsCRP, and ESR) and cytokines (IL-1β, IL-4, TNF-α, IL-6, IL-8, IFN-γ, and IgE) decreased, but not significantly so in most cases (Fig. 4 and Table 2). IL-8, TNF-α, IFN-γ, and IgE levels decreased more in the LP group than in the placebo group. Antioxidant (GPx, SOD) and NOX levels did not change in either group (Fig. 4 and Table 2).

**Table 2:** Comparison between placebo and LP groups.

Outcome	Visit 1 (baseline)			Visit 2 (end of study)			Change from baseline			
	Placebo	LP	*P* value*	Placebo	LP	*P* value*	Placebo	LP	*P* value†	Effect size‡
BCSS score	3.73 (0.65)	4.55 (1.92)	.488	3.09 (1.14)	2.64 (0.92)	.439	−0.64 (1.36)	−1.91 (1.76)	.113	0.345
COAT score	12.09 (4.72)	13.09 (3.65)	.596	8.64 (3.64)	10.36 (5.12)	.338	−3.45 (3.42)	−2.73 (5.50)	.692	0.092
CAT score	14.91 (8.04)	15.45 (7.27)	.510	11.00 (8.88)	11.64 (6.92)	.553	−3.91 (12.34)	−3.82 (6.52)	.947	0.021
FVC	3.15 (0.39)	3.01 (0.41)	.393	3.19 (0.55)	3.02 (0.49)	.470	0.03 (0.30)	0.00 (0.16)	.921	0.028
FEV1	2.56 (0.36)	2.47 (0.38)	.718	2.58 (0.43)	2.42 (0.41)	.393	0.02 (0.25)	−0.05 (0.10)	.533	0.140
FEV1/FVC	81.27 (6.72)	81.82 (3.16)	.947	81.36 (5.16)	80.18 (3.49)	.642	0.09 (3.73)	-1.64 (4.48)	.222	0.267
WBC	5.32 (1.10)	5.95 (1.41)	.293	5.19 (1.22)	5.92 (1.36)	.168	−0.13 (0.93)	−0.03 (0.87)	.646	0.105
hsCRP	2.21 (3.86)	3.54 (6.64)	.793	0.69 (0.59)	0.86 (1.11)	.622	−1.52 (3.47)	−2.68 (6.00)	.599	0.119
ESR	11.00 (7.04)	16.55 (7.42)	.065	8.18 (6.45)	14.09 (9.15)	.066	−2.82 (7.32)	−2.45 (6.83)	1.000	0.000
Eosinophil	2.62 (2.24)	2.71 (1.87)	.844	3.05 (2.23)	2.50 (1.64)	.646	0.43 (1.16)	−0.21 (0.89)	.168	0.301
T-cell	69.56 (8.67)	76.45 (7.37)	.049	72.44 (6.66)	76.45 (8.55)	.224	2.87 (4.97)	0.00 (3.33)	.309	0.224
B-cell	8.15 (3.69)	7.32 (2.08)	.693	6.36 (2.84)	7.10 (2.30)	.264	−1.79 (2.27)	−0.22 (1.54)	.094	0.364
IL-1β	3.13 (1.35)	2.96 (1.10)	.450	3.11 (1.39)	2.87 (1.04)	.511	−0.02 (0.36)	−0.09 (0.47)	.431	0.175
IL-4	25.22 (27.44)	43.31 (103.67)	.509	19.51 (27.70)	42.18 (105.47)	.596	−5.71 (11.08)	−1.13 (3.86)	.355	0.204
TNF-α	14.44 (5.71)	14.40 (4.56)	.767	13.97 (5.28)	13.09 (4.23)	.844	−0.48 (2.77)	−1.31 (1.67)	.264	0.245
IL-6	4.29 (10.41)	2.39 (4.13)	.741	2.23 (4.32)	2.19 (4.29)	.614	−2.06 (6.12)	−0.20 (0.40)	.529	0.141
IL-8	9.85 (6.96)	12.08 (8.87)	.599	10.43 (6.62)	11.03 (7.35)	.948	0.58 (3.47)	-1.05 (4.93)	.599	0.119
IFN-γ	94.78 (65.48)	88.47 (54.51)	.869	94.02 (66.14)	78.37 (52.94)	.325	−0.76 (8.30)	−10.10 (15.61)	.115	0.343
IgE	117.66 (216.03)	216.49 (324.41)	.042	104.92 (192.40)	181.44 (276.62)	.036	−12.75 (24.67)	−35.05 (88.65)	.599	0.119
GPx	114.84 (24.99)	109.75 (18.13)	.574	97.71 (14.01)	91.69 (14.23)	.259	−17.13 (32.48)	−18.06 (29.03)	.693	0.091
SOD	1.21 (0.51)	1.27 (0.47)	.793	1.23 (0.53)	1.09 (0.42)	.669	0.02 (0.49)	−0.19 (0.31)	.293	0.231
NOX	64.48 (49.12)	51.21 (44.66)	.393	53.45 (25.14)	51.08 (28.95)	.646	−11.02 (38.30)	−0.13 (33.88)	.431	0.175

Data are presented as mean (standard deviation).

BCSS = breathlessness, cough, CAT = chronic obstructive pulmonary disease assessment test, COAT = cough assessment test, ESR = erythrocyte sedimentation rate, FEV1 = forced expiratory volume at 1 s, FVC = forced vital capacity, GPx = glutathione peroxidase, hsCRP = high-sensitivity C-reactive protein, IFN-γ = interferon-γ, IgE = immunoglobulin E, IL-1β = interleukin-1β, IL-4 = interleukin-4, IL-6 = interleukin-6, IL-8 = interleukin-8, LP = Liriope platyphylla, MWW = Mann-Whitney-Wilcoxon, NOX = nitric oxide, SOD = superoxide dismutase, TNF-α = tumor necrosis factor-α, WBC = white blood cells.

* MWW rank-sum test.

† Unadjusted MWW rank-sum test.

‡ Cohen *d* effect size for MWW rank-sum test.

One adverse event was reported in the placebo group, when a participant had elevated glucose. When the test was performed again, their levels were in the normal range. No association with LP extract was found, and the laboratory test analysis revealed no hepatotoxicity or nephrotoxicity. There were no significant differences in laboratory test results between the groups (Table 3).

**Table 3:** Laboratory test comparison.

Variable		Observed value			Change from baseline		
		Placebo, n = 11	LP, n = 11	*P* value*	Placebo, n = 11	LP, n = 11	*P* value*
Glucose	Screening	97.73 ± 9.87	99.36 ± 8.1	.68			
	Visit 2	98 ± 13.19	101 ± 13.08	.60	0.27 ± 6.94	1.64 ± 10.95	.73
RBC	Screening	4.55 ± 0.31	4.291 ± 0.25	.05			
	Visit 2	4.54 ± 0.32	4.288 ± 0.26	.06	−0.01 ± 0.13	0 ± 0.15	.86
Hemoglobin	Screening	14.08 ± 1.18	13.19 ± 0.83	.05			
	Visit 2	14.01 ± 1.11	13.15 ± 0.77	.05	−0.07 ± 0.42	−0.04 ± 0.4	.84
Hematocrit	Screening	41.15 ± 2.95	38.94 ± 2.05	.05			
	Visit 2	40.69 ± 2.83	38.75 ± 1.79	.07	−0.46 ± 1.16	−0.18 ± 1.38	.61
Platelet	Screening	231.5 ± 42.96	225.8 ± 39.22	.75			
	Visit 2	223.6 ± 35.92	231 ± 35.33	.63	−7.82 ± 14.43	5.18 ± 15.09	.05
BUN	Screening	14.15 ± 2.02	14.93 ± 4.98	.64			
	Visit 2	13.33 ± 2.56	13.9 ± 3.95	.69	−0.83 ± 3.45	−1.03 ± 4.18	.90
Creatinine	Screening	0.70 ± 0.11	0.68 ± 0.09	.76			
	Visit 2	0.68 ± 0.09	0.70 ± 0.13	.62	−0.02 ± 0.03	0.02 ± 0.05	.03
AST	Screening	23.36 ± 5.94	24.82 ± 5.83	.57			
	Visit 2	24.27 ± 8.22	25.64 ± 6.86	.68	0.91 ± 5.59	0.82 ± 3.63	.96
ALT	Screening	19 ± 12.34	20.82 ± 10.63	.72			
	Visit 2	20.73 ± 13.99	18.55 ± 8.14	.66	1.73 ± 8.36	−2.27 ± 5.29	.19
ALP	Screening	54.82 ± 16.82	61.55 ± 13.95	.32			
	Visit 2	56.73 ± 18.31	63.64 ± 13.99	.33	1.91 ± 6.74	2.09 ± 4.32	.94
Total bilirubin	Screening	0.68 ± 0.15	0.91 ± 0.35	.05			
	Visit 2	0.62 ± 0.15	0.82 ± 0.15	.01	−0.06 ± 0.16	−0.1 ± 0.34	.77
GGT	Screening	20.09 ± 11.61	21.18 ± 26.02	.9			
	Visit 2	19.45 ± 10.45	20.45 ± 22.95	.9	−0.64 ± 4.03	−0.73 ± 3.29	.95
Total cholesterol	Screening	207.5 ± 31.51	188.10 ± 29.77	.15			
	Visit 2	210.5 ± 33.1	192.50 ± 31.33	.21	2.91 ± 15.7	4.36 ± 15.32	.83
Triglyceride	Screening	144.1 ± 109.2	93.27 ± 35.82	.16			
	Visit 2	159.5 ± 121.06	89.73 ± 21.95	.07	15.36 ± 91.86	−3.55 ± 32.39	.53
HDL	Screening	66.73 ± 16.23	68.18 ± 9.55	.80			
	Visit 2	58.36 ± 13.22	58.36 ± 8.38	.99	−8.36 ± 6.65	−9.82 ± 7.04	.62
LDL	Screening	123.1 ± 23.87	109.20 ± 24.07	.19			
	Visit 2	120.4 ± 22.17	111.80 ± 23.24	.39	−2.67 ± 12.67	2.56 ± 10.87	.31
Na	Screening	140.1 ± 1.45	139.70 ± 2.97	.72			
	Visit 2	140.6 ± 1.5	140.50 ± 1.81	.90	0.55 ± 1.86	0.82 ± 2.44	.77
K	Screening	4.56 ± 0.28	4.36 ± 0.25	.08			
	Visit 2	4.44 ± 0.39	4.31 ± 0.29	.39	−0.13 ± 0.37	−0.05 ± 0.34	.59
Cl	Screening	104.3 ± 1.9	104.40 ± 1.57	.90			
	Visit 2	105.1 ± 1.7	104.90 ± 1.38	.79	0.82 ± 1.08	0.55 ± 1.86	.68

ALP = Alkaline phosphatase, ALT = alanine transaminase, AST = aspartate aminotransferase, BUN = blood urea nitrogen, GGT = Gamma-glutamyl transpeptidase, HDL = high-density lipoprotein, LDL = low-density lipoprotein, LP = Liriope platyphylla, RBC = red blood cell.

* *P* values were compared between groups (independent *t*-test).

## 4. Discussion

LP is a traditionally used herbal medicine to treat respiratory diseases, but existing studies only include nonclinical studies. LP regulates chemotaxis of eosinophils and secretion of cytokines involved in pathogenesis of asthma,^[7]^ inhibits bleomycin-induced pulmonary fibrosis,^[8]^ and inhibits lipopolysaccharide-induced lung injury.^[9]^ This pilot clinical trial explored the efficacy and safety of LP for improving respiratory function in healthy individuals without underlying disease based on the results of preclinical study. The effect size and the sample size required for the main clinical trials were calculated, and the feasibility was confirmed.

At screening, 25 people volunteered for this clinical trial. Two did not satisfy the criteria, and 1 declined to participate, so 22 participants were registered and randomly assigned 1:1 to the LP and placebo groups. None of the participants dropped out. Participants took 1 capsule (500-mg LP extract or dextrin) twice a day for 4 weeks. There were no differences at baseline between the groups (Table 1). Statistical analyses were performed on the FAS population.

The primary outcome, BCSS score, tends to decrease in both placebo and LP groups (Fig. 2A). Subjects were healthy individuals with respiratory dysfunction but without underlying disease, so their symptoms seemed to have improved according to natural progress in placebo group. However, the change in BCSS score at the end of the trial differed significantly between the groups (Fig. 2B). For the BCSS score, the interaction between group and visit was statistically significant, according to the analyses of variance test of the repeated-measures mixed-effects model. This decrease in BCSS score indicates that LP extract may improve respiratory function (Table 2). According to the statistical analysis, Cohen *d* effect size was 0.807, so the estimated necessary sample size for the planned main clinical trial is 50 (with a significance level of 0.05 and power of 0.8). The formula for required sample size for each group in a two-group parallel design is as follows: *n* = (2*(*Z*_(1 − *a*/2) + *Z*_(1 − *b*))^2^)/*eta*^2^. In this formula, *eta* is the effect size; *Z_(.*) is the *(.)×* 100% quartile of the normal distribution; *a* is the significance level (type I error); *b* is the type 2 error; and (1 − *b*) × 100% is power. The result of this calculation for this study is 24.10, yielding a sample size of 25 after rounding up. The total sample size, without considering the dropout rate, would therefore be 50.

FVC is the amount of air exhaled with maximum effort. FEV1 is the amount of air exhaled during the first second. The ratio of FEV1/FVC is an indicator of lung function and is normally over 70%.^[10]^ The COAT is a questionnaire that scores the degree of coughing and phlegm symptoms using 5 questions.^[11]^ The CAT is a questionnaire that serves as a subjective indicator of dyspnea.^[12]^ There were no significant changes in these indicators (Fig. 3). The level of inflammation was confirmed by analyzing peripheral blood immune indices such as white blood cell count, hsCRP levels, ESR, eosinophil count, T cell count, and B cell count. We also analyzed cytokine levels (IL-1β, IL-4, TNF-α, IL-6, IL-8, IFN-γ, and IgE) to confirm the degree of inflammation as a secondary outcome. The immune indices did not differ significantly between the groups, and neither did the cytokine levels, although the IL-8, TNF-α, and IFN-γ levels decreased more in the LP group than in the placebo group (Fig. 4). By analyzing antioxidants such as GPx, SOD, and NOX, we showed that LP extract had no significant relationship with oxidation reactions (Fig. 4). Subjects in this clinical trial were healthy individuals, so those with PFT problems or underlying diseases were excluded according to the criteria. Secondary outcomes were based on the Republic of Korea’s Guidance on New Functional Evaluation of Health Functional Foods. Secondary outcomes were PFT, cytokines, and antioxidants, so most of the healthy individuals were normal, and the number of subjects might not be enough to confirm significant changes. Nevertheless, the LP group tended to reduce the expression of inflammatory cytokines.

There was 1 adverse event, which involved elevated glucose levels, but was not related to the clinical trial. Laboratory tests showed no hepatotoxicity or nephrotoxicity for LP extract (Table 3). Consuming LP extract at a dose of 1000 mg/d is considered to be safe for adults.

The validation variables in the FAS population were improved via the consumption of LP extract. The BCSS score decreased more in the LP group than in the placebo group, and cytokine levels decreased somewhat in the LP group. These results suggest that LP extract is anti-inflammatory and improves respiratory function. There were no serious adverse events during the trial. Since this was a pilot trial, the number of participants was insufficient to derive significant results. However, enough evidence has been obtained to justify future larger clinical trials. The significance of the efficacy and safety of LP extract should be evaluated in further research.

## 5. Conclusion

LP extract can alleviate respiratory symptoms in healthy individuals, and it is safe to be taken as supplements. The statistical analysis for future main clinical trial was performed, and the necessary sample size was calculated.

## Author contributions

All authors in the article participated in the clinical trial design and reviewed the article. ESW acquired the data during the clinical trial and wrote the article. YHK and HL performed the trial, analyzed data, and reviewed the article. ENL and IWK conducted a basic study and reviewed the article. JHK supervised the clinical trial and edited the article.
